# Antitumor Activity of a Novel Oncrasin Analogue Is Mediated by JNK Activation and STAT3 Inhibition

**DOI:** 10.1371/journal.pone.0028487

**Published:** 2011-12-12

**Authors:** Wei Guo, Shuhong Wu, Li Wang, Xiaoli Wei, Xiaoying Liu, Ji Wang, Zhimin Lu, Melinda Hollingshead, Bingliang Fang

**Affiliations:** 1 Department of Thoracic and Cardiovascular Surgery, The University of Texas MD Anderson Cancer Center, Houston, Texas, United States of America; 2 Department of Neuro-Oncology, The University of Texas MD Anderson Cancer Center, Houston, Texas, United States of America; 3 Biological Testing Branch, The National Cancer Institute, Frederick, Maryland, United States of America; Bauer Research Foundation, United States of America

## Abstract

**Background:**

To optimize the antitumor activity of oncrasin-1, a small molecule identified through synthetic lethality screening on isogenic K-Ras mutant tumor cells, we developed several analogues and determined their antitumor activities. Here we investigated *in vitro* and *in vivo* antitumor activity of NSC-743380 (1-[(3-chlorophenyl) methyl]-1H-indole-3-methanol, oncrasin-72), one of most potent analogues of oncrasin-1.

**Methodology and Principal Findings:**

*In vitro* antitumor activity was determined in NCI-60 cancer cell line panel using cell viability assay. *In vivo* antitumor activity was determined in parallel with NSC-741909 (oncrasin-60) in xenograft tumors established in nude mice from A498, a human renal cancer cell line. Changes in gene expression levels and signaling pathway activities upon treatment with NSC-743380 were analyzed in breast and renal cancer cells by Western blot analysis. Apoptosis was demonstrated by Western blot analysis and flow cytometric analysis. NSC-743380 is highly active against a subset of cancer cell lines derived from human lung, colon, ovary, kidney, and breast cancers. The 50% growth-inhibitory concentration (GI_50_) for eight of the most sensitive cell lines was ≤10 nM. *In vivo* study showed that NSC-743380 has a better safety profile and greater antitumor activity than NSC-741909. Treatment with NSC-743380 caused complete regression of A498 xenograft tumors in nude mice at the tested doses ranging from 67 mg/kg to 150 mg/kg. Mechanistic characterization revealed that NSC-743380 suppressed the phosphorylation of C-terminal domain of RNA polymerase II, induced JNK activation, inhibited JAK2/STAT3 phosphorylation and suppressed cyclin D1 expression in sensitive human cancer cells. Blocking JNK activation or overexpression of constitutively active STAT3 partially blocked NSC-743380-induced antitumor activity.

**Conclusions:**

NSC-743380 induces antitumor activity through modulation of functions in multiple cancer related pathways and could be a potential anticancer agent for some solid tumors.

## Introduction

Synthetic lethality screening has recently been used by various investigators to identify genes that are crucial for survival of certain oncogene-transformed cells [1], [2] or that sensitize cells to chemotherapy [3], or small molecules that selectively induce cell death in a subset of oncogene-transformed cells [4]–[6]. The concept of synthetic lethality can be defined as a lethal phenotype elicited by two events or mutations in two genes. For example, mutations in an oncogene may render the cell vulnerable to a functional change in another gene. Similarly, if a cell line contains a mutation in one oncogene, synthetic lethality can be elicited by a small molecule that induces or mimics biological functions of a synthetic lethal mutation in the second gene. Thus, synthetic lethality screening allows us to identify cytotoxic agents that are lethal in cancer cells but not in isogenic normal counterparts.

Using synthetic lethality screening on isogenic cell lines with or without a mutant K-*Ras* gene, we recently identified a small molecule (designated oncrasin-1) that kills immortalized and tumorigenic human ovarian epithelial cells expressing mutant K-*Ras* but not cells expressing wild-type *Ras* genes [6]. To improve antitumor activity of a compound identified through the screening on a chemical library, we developed and tested several oncrasin-1 analogues. A few of them were found to be more active than oncrasin-1 in sensitive cancer cells and were further evaluated for their anticancer activities in NCI-60 cancer cell line panel. One of these, NSC-741909 (1-[(4-chlorophenyl) methyl]-1H-indole-3-methanol, oncrasin-60), was found to suppress the growth of several NCI-60 cancer cell lines with a unique anticancer spectrum [7]. Mechanistic studies by reverse-phase protein microarray (RPPA) revealed that treatment with NSC-741909 led to sustained elevation of MAP kinase (P38 MAPK, ERK, and JNK) phosphorylation by suppressing their dephosphorylation. Inhibition of JNK by its specific inhibitor or by dominant negative constructs partially diminished NSC-741909–induced cell killing, indicating that sustained activation of JNK induced by NSC-741909 contributed to NSC-741909–mediated cell death [7].

NSC-743380 (1-[(3-chlorophenyl) methyl]-1H-indole-3-methanol, oncrasin-72) is another potent analogue we identified during analogue analysis for oncrasin-1. Here we report the *in vitro* activity of NSC-743380 in the NCI-60 cancer cell line panel and the *in vivo* activities of NSC-743380 and NSC-741909 in human xenograft tumors in nude mice. NSC-743380 has similar anticancer spectrum and activity as NSC-741909 in the NCI-60 cell line panel. Interestingly, NSC-743380 has much better *in vivo* activity and safety profiles than NSC-741909. Therefore, we further investigated the mechanisms of NSC-743380-induced antitumor activity. Our results showed that NSC-743380 may induce antitumor activity by modulating the functions of multiple cancer-related targets or pathways.

## Results

### Antitumor Activity of NSC-743380 in NCI-60 Cancer Cell Line Panel

**Figure 1:**
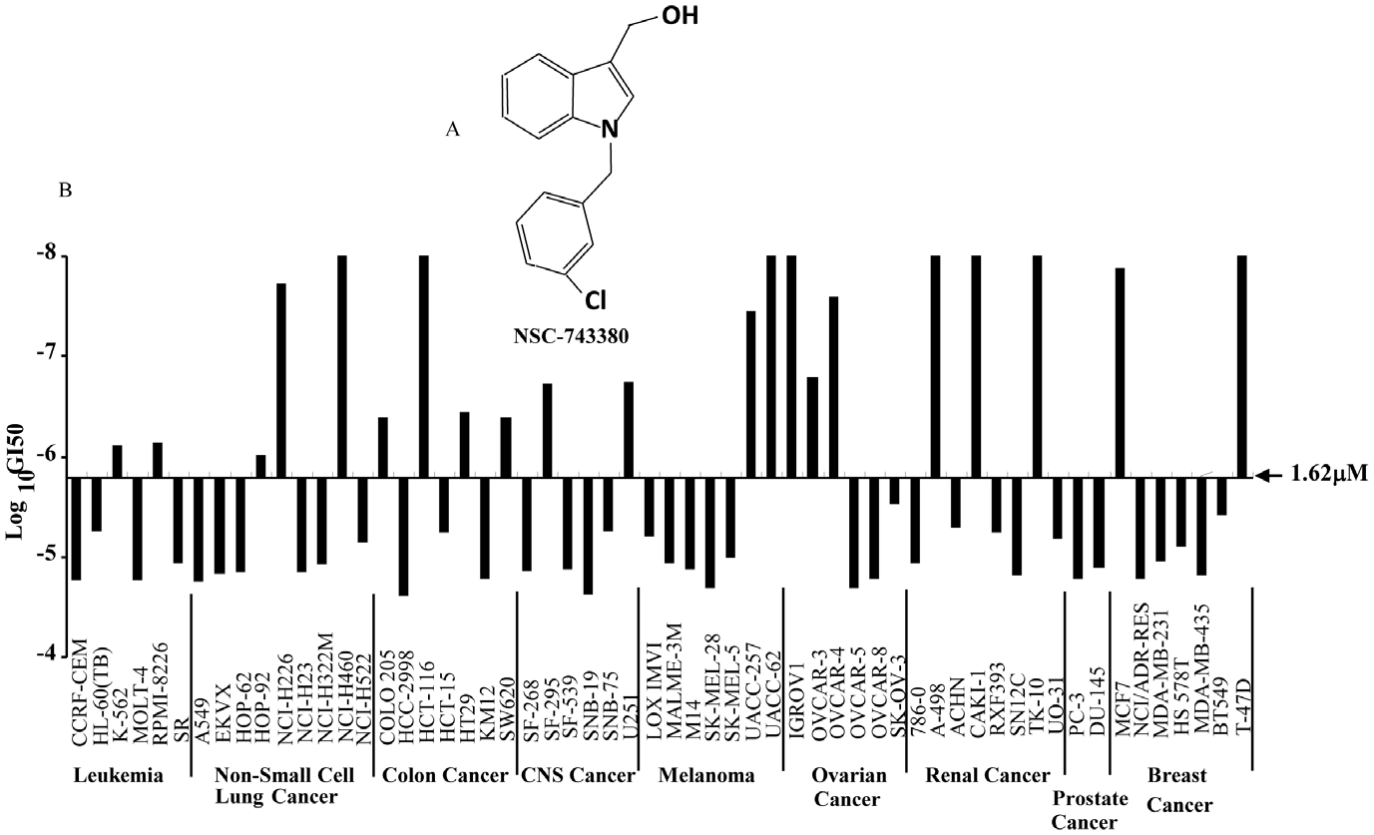
Antitumor activity of NSC-743380 in the NCI-60 cancer cell line panel. A) The structure of NSC-743380. B) The 50% growth-inhibitory concentrations (GI_50_) at logarithmic scale were calculated and are shown in the bar graph. The middle line represents the median GI_50_ for NSC-743380 in NCI-60 cancer cell line panel.

To optimize the compound we identified through synthetic lethality screening, we evaluated several analogues with chemical structures similar to that of oncrasin-1. We previously reported the antitumor activity of an analogue, NSC-741909, in NCI-60 cancer cell lines [7]. NSC-743380, another analogue with close structural similarity to NSC-741909, was also evaluated in the NCI-60 cell line panel. The only difference in chemical structure is that the chlorine in NSC-741909 is in the p-position, whereas in NSC-743380 it is in the n-position (Fig. 1). The tests performed by the Developmental Therapeutics Program at the NCI showed that, like NSC-741909, NSC-743380 is highly active against various cancer cell lines from the lung, colon, ovary, kidney, and breast. In 58 of the 60 cancer cell lines tested, the median 50% growth-inhibitory concentration (GI_50_) for NSC-743380 was 1.62 µM. For eight of the most sensitive cell lines, the GI_50_ was ≤10^−8^ M (10 nM), the lowest concentration tested by NCI (Fig. 1). The anticancer spectrum and activity in the NCI-60 cell line panel are very similar for NSC-741909 and NSC-743380.

### *In Vivo* Activity of NSC-743380 and NSC-741909

**Figure 2:**
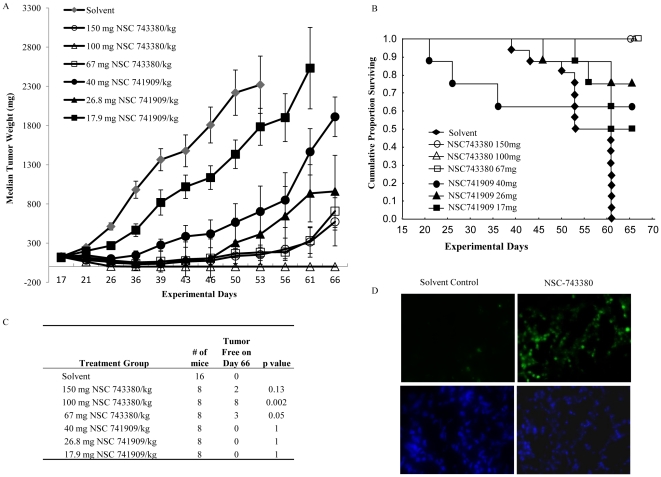
Effect on subcutaneous A498 renal tumor xenografts. **A**) Median tumor weights of A498 human renal tumor xenografts in mice receiving vehicle control, NSC-743380 or NSC-741909 treatment. Data are presented with the standard errors of the median shown as error bars. Each treatment group comprised 8 mice, and the control group 16. All treatment groups were statistically significantly different (Student's *t* test) from the control at multiple time points. B) Kaplan Meier Survive Curve of the animals shown in A. C) The numbers of tumor-free mice at day 66. D) TUNEL and DAPI staining of tumors treated with solvent or NSC-743380 (100 mg/kg).

A comparison analysis of the antitumor spectrum of NSC-741909 in NCI-60 cell lines with those of well-studied anticancer agents showed that NSC-741909 lies outside the category of adequately studied classes of antitumor agents, suggesting that NSC-741909 and NSC-743380 may have novel anticancer mechanisms. We therefore performed *in vivo* activity tests on these two compounds in a subcutaneous tumor model established from A498, a renal cancer cell line, through collaboration with the Developmental Therapeutics Program at NCI. An initial dose tolerance test showed that, although NSC-743380 and NSC-741909 have very close structural similarity, they have quite different in *in vivo* tolerance levels. The maximum tolerated intraperitoneal doses in mice for NSC-741909 and NSC-743380 on a daily 5 times schedule were 40 mg/kg and 150 mg/kg, respectively. In the *in vivo* activity studies, therefore, NSC-741909 and NSC-743380 were given at different dose levels. NSC-741909 was administered at doses ranging from 17.9 mg/kg to 40 mg/kg, whereas NSC-743380 was administered at doses ranging from 67 mg/kg to 150 mg/kg. Treatment with NSC-743380 caused tumor regression at all three dose levels, and treatment with NSC-741909 produced tumor regression or stabilization at the high and intermediate dose levels (Fig. 2). Whereas all animals in the control group were sacrificed due to tumor burden by day 61, all mice treated with different doses of NSC-743380 survived at day 66 (Fig. 2B). A substantial number of animals treated with NSC-743380 at all dose levels remained tumor free on day 66 (Fig. 2C). No obvious toxic effects were observed in animals treated with NSC-743380; however, some weight loss occurred in animals treated with the high doses of NSC-741909 (Fig. S1). This result not only demonstrates the *in vivo* activity of both compounds, it also suggests that compounds of similar chemical structures with similar *in vitro* activities may have dramatically different *in vivo* activities and tolerances, highlighting the importance of optimizing *in vivo* activities. Terminal deoxynucleotidyl transferase dUTP nick end labeling (TUNEL) assay revealed a remarkable apoptosis in tumors harvested from NSC-743380 treated mice (100 mg/kg of for 3 days), but not in tumors from solvent treated mice (Fig. 2D), demonstrating effective *in vivo* apoptosis induction in xenograft tumor tissues by NSC-743380.

### Induction of Apoptosis by NSC-743380

**Figure 3:**
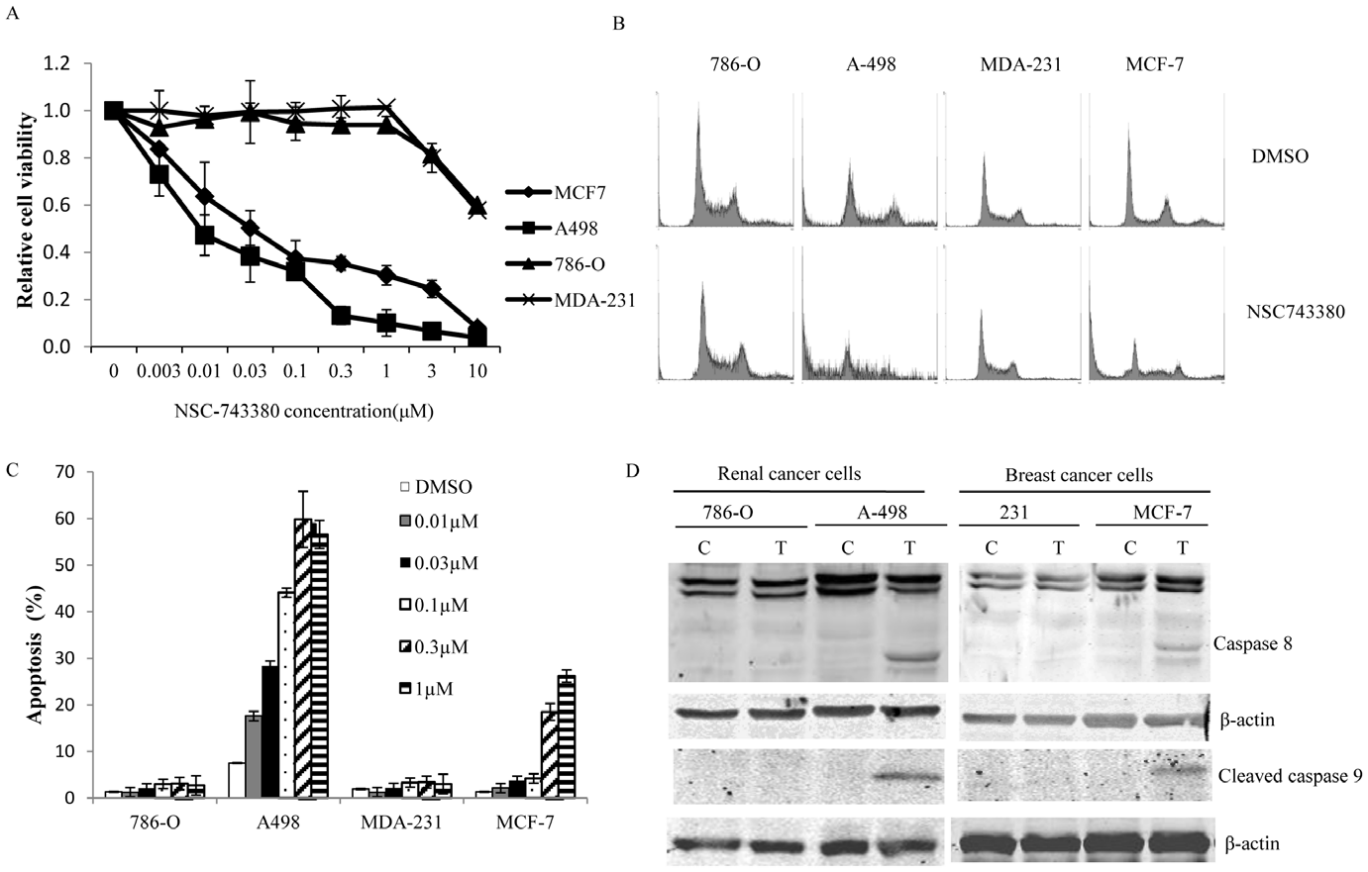
Induction of apoptosis by NSC-743380. Two renal cancer cell lines (786-O and A498) and two breast cancer cell lines (MCF-7 and MDA-MD-231) were treated with different doses of NSC-743380. Cells treated with DMSO were used as controls. Cell viability was assayed 72 h after treatment by the SRB method. Apoptotic cells were determined by flow cytometric analysis 24 h after treatment. A) Cell viability assay. Viability of control cells was set as 1. Each data point represents the mean ± SD of three independent experiments. B) Histogram of cell cycle analysis at 24 h after treatment with 1 µM NSC-743380. C) Percentages of apoptotic cells. The values represent the means ± SD of three analyses. D) The four cell lines were treated with 1 µM NSC-743380 for 12 h. Whole-cell lysates were harvested for Western blot analysis of caspase-8 cleavage and cleaved caspase 9. C: controls; T: treated with NSC-743380.

The promising *in vivo* activity of NSC-743380 in the A498 tumor xenograft model prompted us to further investigate the mechanisms of NSC-743380–induced antitumor activity in A498 cells. To determine whether the changes detected in A498 cells were also applicable to other sensitive cells, we included MCF-7 cells in our studies. We also included 786-O and MDA-MB-231 cell lines that were resistant to NSC-743380 treatment in order to detect differences in response between sensitive and resistant cells. NSC-743380–induced dose-dependent growth inhibition was evaluated in all four cell lines (two breast cancer cell lines and two renal cancer cell lines). The results showed the IC_50_ for MCF-7 and A498 were 0.02 µM and 0.01 µM, respectively, whereas the IC_50_ for 786-O and MDA-MB-231 were higher than 10 µM (the highest concentration we tested) (Fig. 3A).

To determine whether the antitumor activity of NSC-743380 is due to induction of apoptosis or growth inhibition, we performed cell cycle analysis on the same four cell lines after treatment with NSC-743380 (0.01–1 µM) (Fig. 3B). The sub-G_1_ populations were increased in a dose-dependent manner in A498 and MCF-7 cells (Fig. 3C). In contrast, there was no significant change in sub-G_1_ populations in 786-O and MDA-MB-231 cells after NSC-743380 treatment. This indicates that NSC-743380 can effectively induce apoptosis in sensitive cells. Western blot analysis showed that treatment with 1 µM NSC-743380 effectively activated caspase-8 and caspase 9 in MCF-7 and A498 cells (Fig. 3D), indicating that its cytotoxic effect in cancer cells is due to its induction of apoptosis. No cleaved caspase-8 or caspase-9 could be detected in resistant 786-O and MDA-MB-231 cells at 1 µM NSC-743380.

### NSC-743380 Modulates Functions of Multiple Cancer-Related Pathways

**Figure 4:**
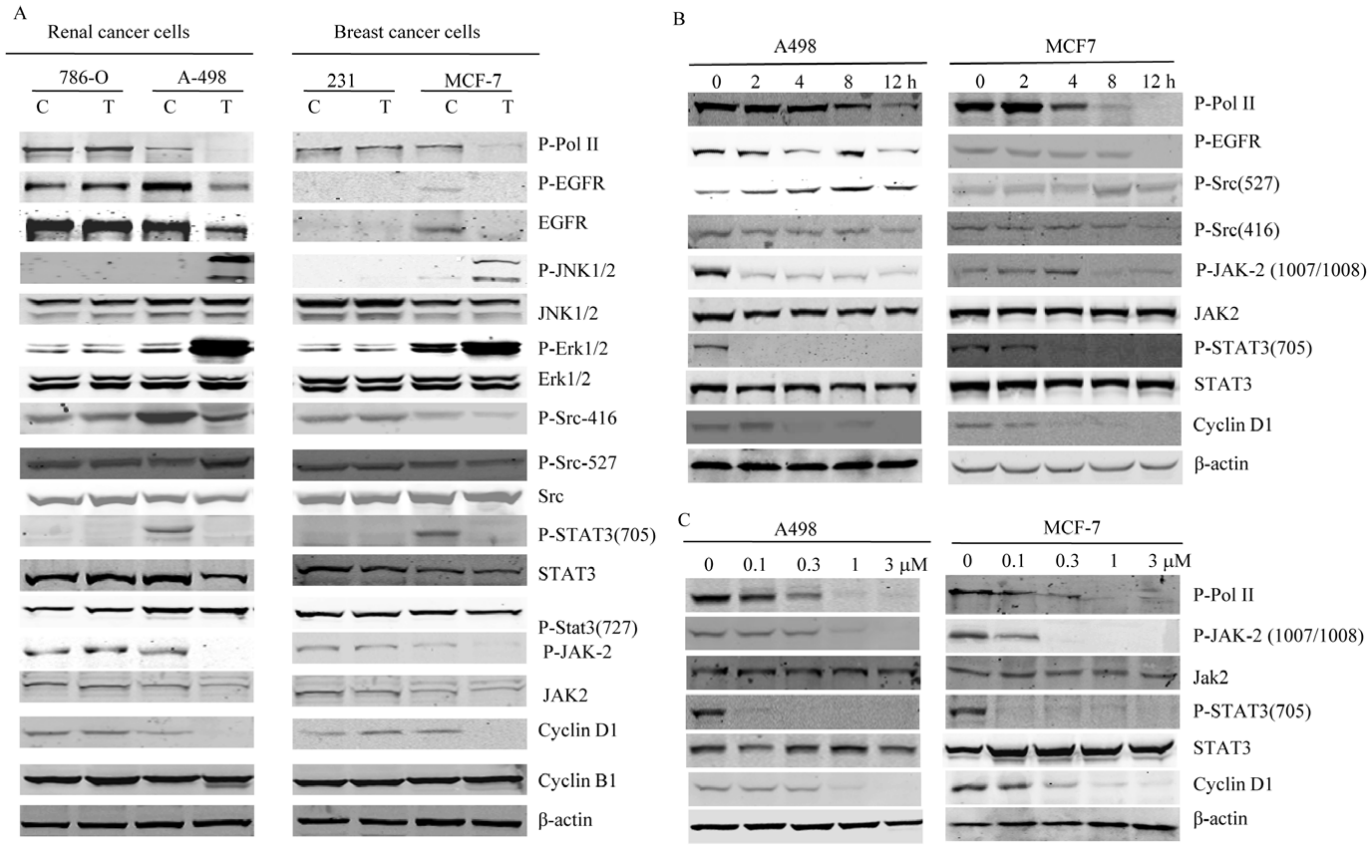
Modulating functions of multiple pathways by NSC-743380. A) Changes in molecular markers of various pathways after treatment with 1 µM NSC-743380 for 12 h in breast and renal cancer cells. C: controls; T: cells treated with NSC-743380. B) Time-dependent changes in p-EGFR, p-Src, p-JAK2, and p-STAT3 in A498 and MCF-7 cells after treatment with 1 µM NSC-743380. C) Dose-dependent changes in p-EGFR, p-Src, p-JAK2, and p-STAT3 in A498 and MCF-7 cells after treatment with different doses of NSC-743380 for 12 h.

To further determine molecular mechanisms of NSC-743380 induced antitumor activity, we investigated effects of NSC-743380 on phosphorylation of RNA polymerase II and various cancer-related pathways, including basal and phosphorylated levels of growth factor receptors, Src tyrosine kinase, JNK and ERK1/2, STAT3, JAK2, cyclin B1 and cyclin D1, in sensitive and resistant cells after treatment with 1 µM NSC-743380 for 12 h. The results show that, like the leader compound oncrasin-1 [8], NSC-743380 could effectively inhibit phosphorylation of RNA polymerase II (Fig. 4A). Moreover, the levels of phosphorylated JNK (Thr181/Tyr185) and ERK1/2 (Thr202/Tyr204) were increased in sensitive cells after treatment, but not in resistant cells. Furthermore, the levels of phospho-EGFR (Tyr1068), phospho-Src (Tyr416), phospho-STAT3 (Tyr705), JAK2 (Try1007/1008) and cyclin D1 were decreased in sensitive cells but not in resistant cells. No significant changes were observed in cyclin B, total JNK, Erk, Src, STAT3, or JAK2 after NSC-743380 treatment. This result suggested that treatment with NSC-743380 induced functional changes in multiple pathways, including mRNA elongation/processing, EGFR, JNK, Erk, Src, and JAK2/STAT3 pathways.

We then tested the time dependency of the above molecular changes. Cells were treated with 1 µM NSC-743380 for 2–12 h, and cell lysates were subjected to Western blot analysis. The results showed that the decrease in phosphorylation of JAK2 and STAT3 occurred relatively earlier (2–4 h) than the decreases in Src phosphorylation, and phosphorylated EGFR, suggesting that inhibition of JAK2 and STAT3 (Y705) might be Src- or EGFR-independent (Fig. 4B). The decrease of cyclin D1 and phosphorylated RNA polymerase II occurred at 4–8 h after the treatment. We also evaluated the dose effect on RNA polymerase II, JAK2/STAT3 and cyclin D1 in two sensitive cell lines and found that cyclin D1 and the phosphorylation of RNA polymerase II, JAK2 and STAT3 were suppressed in a dose-dependent manner (Fig. 4C). Together, these data suggest that NSC-743380 modulating multiple targets in signaling pathways at time- and dose-dependent manners.

**Figure 5:**
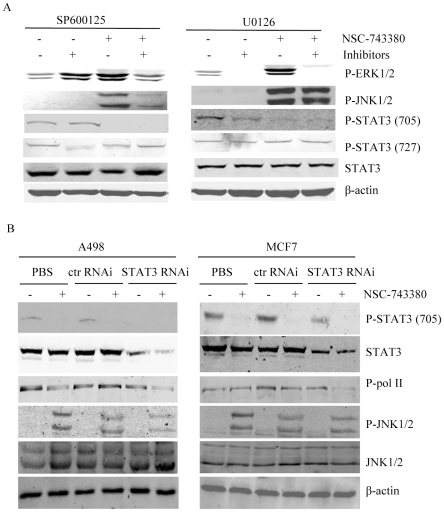
Independent effects on JNK, STAT3 and RNA polymerase II induced by NSC-743380. A) Effect of JNK and ERK inhibitors. MCF-7 cells were treated 1 µM NSC-743380 in the presence or absence of 10 µM SP600125 or 10 µM U0126 and examined for expression of p-ERK, p-JNK, and p-STAT3 (Tyr705 and Ser727). Whole-cell lysates were subjected to Western blot analysis, and β-actin was used as the loading control. B) Effect of STAT3 knockdown. After transfected with control RNAi or STAT3 RNAi for 48 hours, A498 and MCF7 were treated with 1 µM NSC-743380 for 6 hours. Phosphorylation of JNK, STAT3 and PloII were determined by Western Blot analysis. β-actin was used as the loading control.

Because MAPK pathway activation was reported to inhibit STAT3 phosphorylation at site Tyr705 [9] and NSC-743380 induced dramatic increases in pJNK and pERK, we tested whether pretreating MCF7 cells with the JNK inhibitor II (SP600125) or a MEK inhibitor (U0126) at 10 µM for 2 h would block NSC-743380–induced inhibition of STAT3 phosphorylation at site Tyr705. The results showed that blocking NSC-743380–induced JNK and ERK activation did not affect NSC-743380–mediated STAT3 suppression (Fig. 5A), suggesting that NSC-743380-induced STAT3 inhibition is not associated with MAPK activation. We also investigated whether suppression of STAT3 could have impact on JNK activation or RNA polymerase II suppression. For this purpose, we treated A498 and MCF-7 cells with STAT3 siRNA and determined the effect on JNK activation and RNA polymerase II suppression. Knockdown of STAT3 alone did not induce JNK activation and had minimal effect on RNA polymerase II phosphorylation (Fig. 5B), suggesting that NSC-743380-induced effects on JNK, STAT3 and RNA polymerase II could be independent.

### JNK inhibitor or Constitutively Active STAT3 Partially Diminished NSC-743380-induced Cell Killing

**Figure 6:**
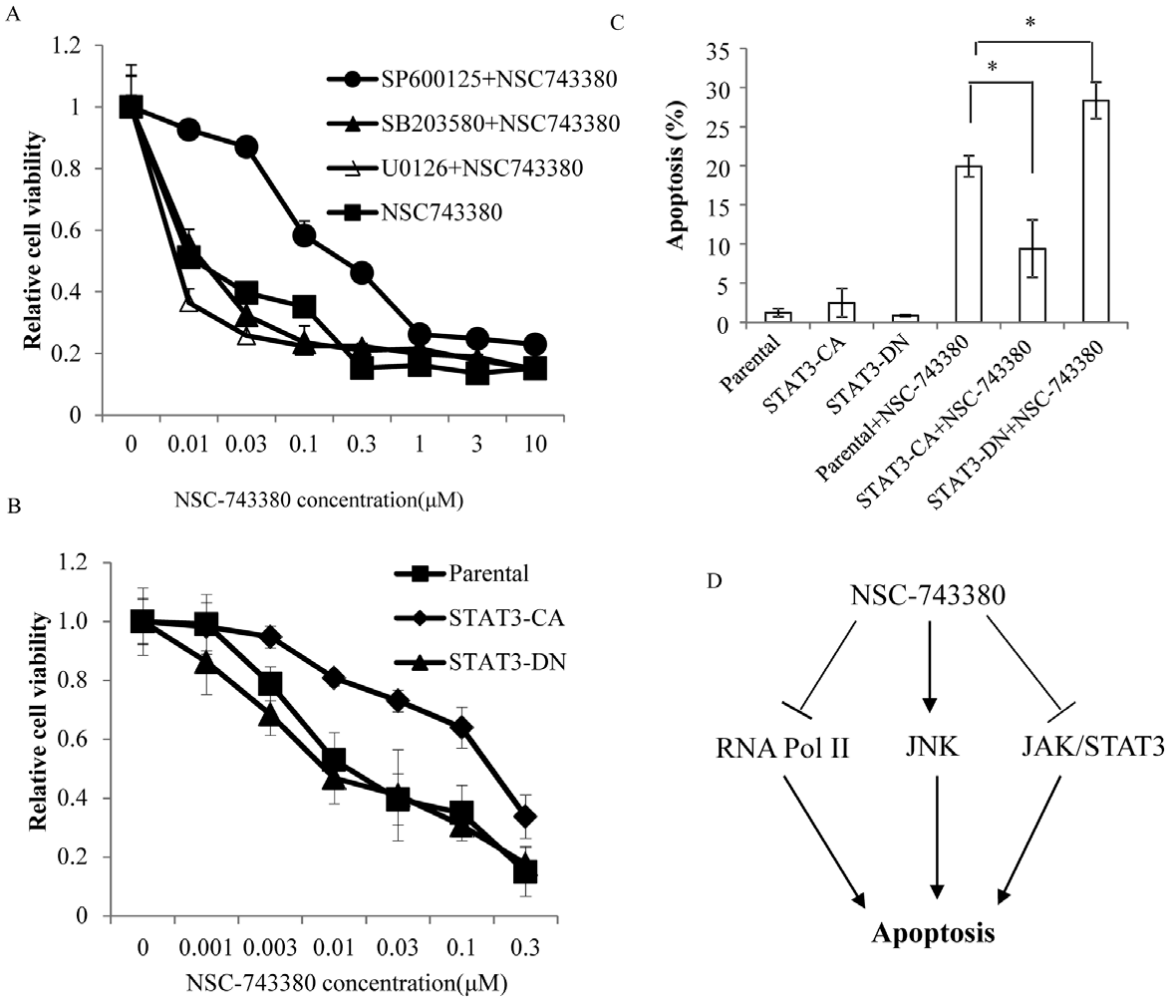
Effect of JNK inhibitor and constitutive STAT3 on NSC-743380-induced cell killing. A) A498 cells were pretreated with 10 µM of JNK inhibitor SP600125, p38 MAP kinase inhibitor SB203580 or Erk inhibitor U0126 for 2 hours, and then analyzed for their susceptibility to NSC-743380. Dose response to NSC-743380 was determined by the SRB assay. Values represent the means ± SD from three independent experiments. B) A498 cells were transfected with a plasmid expressing STAT3-CA or expressing STAT3-DN. After selection with G418, cells expressing STAT3-CA or STAT3-DN were pooled together. Parental, STAT3-CA–transfected, or STAT3-DN–transfected A498 cells were then analyzed for their susceptibility to NSC-743380. Dose response to NSC-743380 was determined by the SRB assay. C) Percentage of apoptotic cells in parental and STAT3-CA or STAT3-DN transfected A498 cells at 12 h after treatment with 1 µM NSC-743380. * represents a *P* value less than or equal to 0.05. D) Putative mode of action by NSC-743380.

To investigate the role of JNK activation and STAT3 inhibition in NSC-743380–induced apoptosis, we determined that the cell killing effect of NSC-743380 in A498 cells after cells were pretreated with a JNK inhibitor SP600125 [10] or a constitutively active STAT3. NSC-743380 induced cell growth inhibition was partially reversed at the presence of JNK inhibitor SP600125, but was not affected by the presence of p38 MAP kinase inhibitor SB203580 or Erk inhibitor U0126 (Fig. 6A), suggesting that inhibiting JNK activation could partially reverse the antitumor activity of NSC-743380. Stably transfected A498 cells with constitutively active STAT3 (STAT3-CA) but not dominant negative STAT3 (STAT3-DN) also partially reverses NSC-743380 induced antitumor activity (Fig. 6B). The IC_50_ for NSC-743380 in STAT3-CA A498 cells increased to 0.15 µM, whereas IC_50_ in parental cells was 0.01 µM. We also performed fluorescence-activated cell sorting analysis to test the percentage of apoptotic cells and cell viability assay 12 h after treatment with 1 µM NSC-743380 in A498 cells that were stably transfected with STAT3-DN or STAT3-CA constructs (Fig. 6C, and Fig. S3). The result showed STAT3-CA diminished NSC-743380 induced apoptosis whereas STAT3-DN increased the apoptosis. This result suggested that both JNK activation and STAT3 inhibition contributed to NSC-743380 induced antitumor activity and that functional reversal of either pathway could only partially affect NSC-7433380's anticancer activity.

## Discussion

Our results demonstrate that NSC-743380 has potential *in vitro* and *in vivo* antitumor activities and that NSC-743380 modulates functions of multipathways, including inhibiting RNA polymerase phosphorylation, activating MAP kinase JNK, and inhibiting JAK/STAT3 pathway, all of which play critical roles in anticancer therapy and/or tumorigenesis. Although the two sensitive cell lines used in the mechanistic study have wild type p53 while the two resistant cell lines have mutant p53, the status of p53 gene mutation is likely not a critical factor in NSC-743380 induced apoptosis, because a number of p53 mutant cancer cell lines in NCI-60 panel were highly susceptible to NSC-743380. For example, renal cancer cell line TK-10 and breast cancer cell line T-47D are both p53 mutant and highly sensitive to NSC-743380 (Fig. 1).

Evidence has indicated that oncogene-transformed cells require continuous activity of RNA polymerase II to prevent oncogene-induced apoptosis [11]. Anticancer agents such as the pan-cyclin dependent kinase (CDK) inhibitors flavopiridol and seliciclib elicit their antitumor activity by inhibiting RNA polymerase II [12]–[14], leading to suppression of the expression of short-lived antiapoptotic proteins such as Mcl-1, XIAP, Bcl-XL, and survivin [15]–[17], suggesting that RNA polymerase II may serve as a therapeutic target for cancers. The effect of NSC-743380 on RNA polymerase II phosphorylation suggests that it may affect mRNA transcription in general. Indeed, mRNA levels of cyclin D1 and cyclin B1 were both reduced in sensitive cells after NSC-743380 treatment (Text S2 and Fig. S2), although there was no detectable changes of cyclin B1 at the protein level. Similarly, JNK is an important mediator of apoptosis caused by various stress stimuli and anticancer agents [18]–[20]. JNK has been reported to suppress Ras transformation through apoptotic elimination of Ras-transformed cells in vivo [21]. Dominant negative mutations in JNK signaling pathway is associated with human cancers [22]. Activation of JNK leads to phosphorylation of Bcl2 and Bcl-XL [18] and accelerates degradation of the NF-kappaB-induced antiapoptotic protein c-FLIP through phosphorylation and activation of E3 ubiquitin ligase Itch [23], facilitating apoptosis induction. The partial reversal of NSC-743380 induced antitumor activity by a JNK-specific inhibitor suggested that NSC-743380 induced antitumor activity is both JNK-dependent and –independent.

Signal transducer and activator of transcription 3 (STAT3) is a key regulator of gene expression and is activated by tyrosine phosphorylation through members of the JAK (Janus kinase) and other tyrosine kinase families in response to a variety of cytokines and growth factors [24]. JAK/STAT3 has been implicated in oncogenesis, invasiveness and metastasis of some types of cancers [25]–[27]. Efforts have been made in developing small molecule inhibitors of STAT3 and JAK for cancer therapy [28]–[30] and some of them showed good *in vitro* and *in vivo* antitumor activities. Our study showed that NSC-743380 suppressed phosphorylation and activation of JAK2/STAT3 and that NSC-743380–mediated inhibition of STAT3 may contribute partially to NSC-743380–induced antitumor activity, because this antitumor activity was partially diminished when sensitive cells were transfected with a constitutively active STAT3.

Our results suggested that NSC-743380 selectively modulates functions of RNA polymerase II, JNK, and JAK/STAT3 pathways in a subset of cancer cells, because the functionality of those molecules was not affected by NSC-743380 in the resistant cancer cell lines. The mechanisms underlining the selectivity of NSC-743380 in cancer cells were not clear. NSC-743380 may not directly inhibit kinases because a test on 300 kinases by Reaction Biology Corporation (Malverin, PA) showed that, at 1 µM concentration, NSC-743380 did not have appreciable inhibitory effect (or more than 25% inhibition) on those tested kinases (data not shown). Nevertheless, our data suggest that NSC-743380–mediated STAT3 inhibition is not associated with JNK activation, because blocking NSC-743380–induced JNK or ERK phosphorylation had no effect on NSC-743380–mediated inhibition of STAT3. Similarly, knockdown of STAT3 had minimal effect on JNK activation and RNA polymerase inhibition. Together, those results suggest that NSC-743380 may modulate functions of multiple cancer related pathways.

The *in vivo* activity studies reported here show that NSC-743380 is highly active against established xenograft tumors derived from A498 renal cancer cells. The favorable *in vivo* activity induced by this compound at doses without obvious toxicity suggests the possibility of translating this compound for clinical evaluation. Interestingly, although NSC-743380 and NSC-741909 have very similar structures and *in vitro* activities, including their anticancer activities in NCI-60 cell lines, they have dramatically different *in vivo* toxicity and activity profiles. These *in vivo* results also highlight the importance of compound optimization *in vivo*.

## Materials and Methods

### Cell Lines

The human breast cancer cell lines (MDA-MB-231 and MCF-7) and human renal cancer cell lines (A498 and 786-O) were grown in Dulbecco's modified Eagle's medium supplemented with 10% fetal bovine serum and 100 µg/mL penicillin-streptomycin (all from Life Technologies, Gaithersburg, MD). All four cell lines used in the mechanistic study were mycoplasma-free when tested by a polymerase chain reaction (PCR)-based Mycoplasma Test [31], [32].

### Chemicals and Antibodies

NSC-743380 (1-[(3-chlorophenyl) methyl]-1H-indole-3-methanol, C_16_H_14_ClNO, MW 271.7) and NSC-741909 (1-[(4-chlorophenyl) methyl]-1H-indole-3-methanol, C_16_H_14_ClNO, MW 271.7) used for *in vivo* study were synthesized by Developmental Therapeutics Program at NCI. NSC-73380 used for *in vitro* study was synthesized as previously described [33] and has purity of ≥98%. The purity was determined by high-performance liquid chromatography (Agilent Technologies 1200 Series) equipped with a C-18 bounded-phase column (Waters, XTerra C18 MS, 3.5 µm, 4.5×50 mm). A gradient elution was performed with acetonitrile and water as a mobile phase and was monitored at 230 nm. The chemical entity of NSC743380 was determined by LC/MS/UV_230_ and ^1^H and ^13^C NMR analyses (Text S1). Electrospray ionization (ESI) MS spectra were recorded with an Agilent LC/MSD Trap XCT Ultra spectrometer. NMR spectra were recorded with Bruker Avance DRX 300 spectrometer.

Inhibitors of JNK (SP600125) and MEK (U0126) were purchased from EMD Chemicals (Gibbstown, NJ). Antibodies to the following proteins were used for Western blot analysis: JNK, phospho-JNK, STAT3, phospho-STAT3, phospho-JAK2, phospho-Src-416, Phospho-src-527, cleaved caspase 9, cyclin B, and cyclin D1 (Cell Signaling Technology, Danvers, MA), caspase-8 (Alexis Biochemicals, Farmingdale, NY), and β-actin (Sigma-Aldrich, St. Louis, MO).

### Cell Viability Assay

Cell viability was determined by the sulforhodamine B (SRB) assay as previously reported [6]. Relative viability of the treated cells was normalized to the DMSO-treated control cells, which were set at 1. Each experiment was performed in quadruplicate and repeated at least three times. The IC_50_ value, a dose that causes a 50% reduction in surviving cells after 72 hours treatment compared with that of control cells, was calculated by using the CurveExpert Version 1.3 program.

### Flow Cytometry Assay

Cells were seeded at a density of 2.5×10^5^ cells/well in six-well plates and allowed to grow overnight. After treatment, cells were harvested with trypsin and washed twice with phosphate-buffered saline solution (PBS). Cells were fixed with 75% ethanol at a density of 1×10^6^ cells/mL overnight. The cell pellets were then harvested and resuspended with propidium iodide (BD Biosciences, San Hose, CA) for 15 min in the dark at room temperature. The cells were analyzed on an EPICS Profile II flow cytometer (Beckman Coulter, Brea, CA) with the Multicycle Phoenix Flow Systems program (Phoenix Flow Systems, San Diego, CA). All experiments were repeated three times.

### Western Blot Analysis

Western blot analysis was performed as we reported previously [8]. Briefly, cells were harvested and subjected to Laemmli's lysis buffer, and the protein concentration was determined by using the Bradford method. Equal amounts of lysate (40 µg) were separated by 10% sodium dodecyl sulfate–polyacrylamide gel electrophoresis and then transferred to Hybond-enhanced chemiluminescence membranes (GE Healthcare Life Sciences, Piscataway, NJ). Membranes were blocked with PBS containing 5% low-fat milk for 1 h and incubated with primary antibodies overnight at 4°C. After three washes with PBS containing 0.1% Tween-20, membranes were incubated with IRDye infrared secondary antibodies for 1 h at room temperature. The membranes were washed with PBS Tween-20 again and detected with the Odyssey Infrared Imaging System (Lincoln, NE). β-Actin was used as a loading control.

### Plasmid Transfection and Stable Cell Lines

The plasmids expressing STAT3-CA or STAT3-DN were kindly provided by Dr. James Darnell [25]. Plasmid or siRNA oligonucleotides transfection was performed as previously described [7]. Control RNAi and STAT3 RNAi were purchased from Dharmacon Products (Lafayette,CO). Stable transfectants were selected with a medium containing 500 µg/mL G418. Plasmid transfection was performed with FuGENE6 reagent (Roche Diagnostics, Indianapolis, IN).

### Animal Experiments

*In vivo* antitumor activity assays were performed by the Experimental Therapeutics Program at the NCI. Six-week-old female athymic nude mice (Animal Production Program, Frederick, MD) were implanted with A498 tumor fragments obtained from serially passaged donor mice. The tumors, initiated by subcutaneous injection of 10^7^ A498 cells (DCTD Repository, Frederick, MD), were at third *in vivo* passage for this study. When the tumors reached approximately 125 mg, the mice were randomized into treatment groups and therapy was initiated. Tumor growth was monitored by caliper measurements, and tumor weights were calculated as: [tumor length in mm×(tumor width^2^ in mm)]/2 = weight in mg [34]. The treatments were administered intraperitoneally once daily for a total of 14 days. The control group (n = 16) received 10% DMSO in 0.09% saline solution with 0.05% Tween-80 in a dose volume of 0.1 mL/10 g body weight. The experimental treatments (n = 8/group) included NSC-743380 at doses of 150 mg/kg, 100 mg/kg, 67 mg/kg. The dosing solutions were prepared fresh daily by dissolving a known weight of test material in 100% DMSO and subsequently diluting the 10× stock into 0.9% saline solution containing 0.05% Tween-80. Tumor growth and animal body weight were monitored overtime. All treatments were administered on an exact body weight basis using a dose volume of 10 mL/kg of body weight. NSC-743380 was evaluated as suspensions as they were not soluble at the concentrations necessary to achieve the desired doses in mice. The mice were monitored for 66 days after treatment began. The results were evaluated as described previously [34] by measuring tumor volumes and counting tumor-free survivors.

### TUNEL assay

Tumors were established as described above and were allowed to grow to a size of 0.5–0.8 cm^3^ before treatment started. Mice bearing A498 tumors were then treated daily with solvent or 100 mg/kg NSC-743380 (n = 3 animals per group) for 3 days. Four hours after the last dose, animals were euthanized, and tumors were resected and covered with cryo-embedding OCT medium. The tissue blocks were stored at −80°C until ready for sectioning. The TUNEL assay was performed following the manufacturer's instructions of a kit from Promega (DeadEndTM Fluorometric TUNEL System). The slides were evaluated under fluorescence microscopy for apoptotic cells with a strong green nuclear fluorescence. Nucleus staining with DAPI which exhibits a blue nuclear fluorescence was used an indicator for the presence of cells.

### Statistical Analysis

Differences between the treatment groups were assessed by ANOVA and followed by student t test using the software StatSoft. The data on tumor-free survivors were evaluated by Fisher exact test comparing the treated groups to vehicle control groups on day 66. All tests were two-tailed. P-values less than 0.05 were considered statistically significant.

## Supporting Information

Figure S1 **Body weights of mice treated with NSC-743380 or NSC-741909.** Data presented are the mean body weights of mice shown in Figure 2. The mice bearing subcutaneous A498 renal tumor xenografts were treated with vehicle control, NSC-743380 or NSC-741909 at the doses as indicated. Each treatment group had 8 mice while the control group 16 mice.
(TIF)

Figure S2 **mRNA levels of Cyclin D1 and Cyclin B1 expression after treatment with NSC-743380.** A) Time dependent and dose response changes of Cyclin D1 mRNA after treatment with NSC-743380 in A498 cells. B) Dose dependent changes of Cyclin B1 mRNA after treatment with NSC-743380 in A498 cells. The mRNA levels were normalized with that of GAPDH.
(TIF)

Figure S3 **Cell viability of A498, A498 STAT3-CA and A498 STAT3-DN cells after treatment with 1 µM NSC-743380 for 12 h.** Cells treated with DMSO were used as controls and set as 1. Cell viability was assayed 12 h after treatment by the SRB method. Each data point represents the mean ± SD of three independent experiments.
(TIF)

Text S1 **Method for synthesis, purity determination and structure identification of NSC-743380.**
(DOCX)

Text S2 **Quantitative PCR for mRNA analysis.**
(DOCX)
