# Supplementary figures and images for: Antitumor Activity of a Novel Oncrasin Analogue Is Mediated by JNK Activation and STAT3 Inhibition
Source: PLoS One. 2011 Dec 12;6(12):e28487. doi: 10.1371/journal.pone.0028487 (PMC3236185; doi:10.1371/journal.pone.0028487)

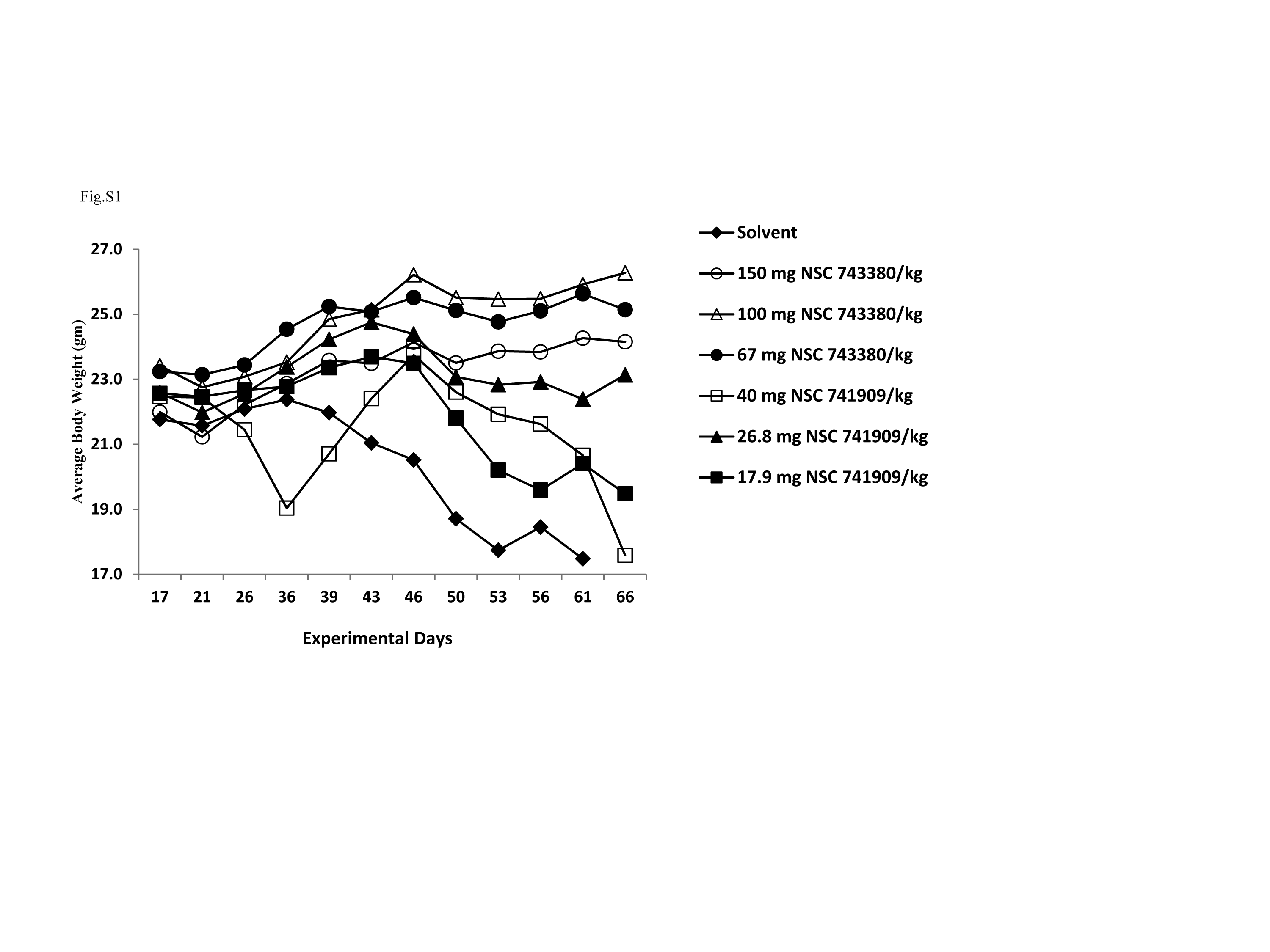

Supplement: Figure S1 — Body weights of mice treated with NSC-743380 or NSC-741909. Data presented are the mean body weights of mice shown in Figure 2. The mice bearing subcutaneous A498 renal tumor xenografts were treated with vehicle control, NSC-743380 or NSC-741909 at the doses as indicated. Each treatment group had 8 mice while the control group 16 mice. (TIF) [file pone.0028487.s001.tif]

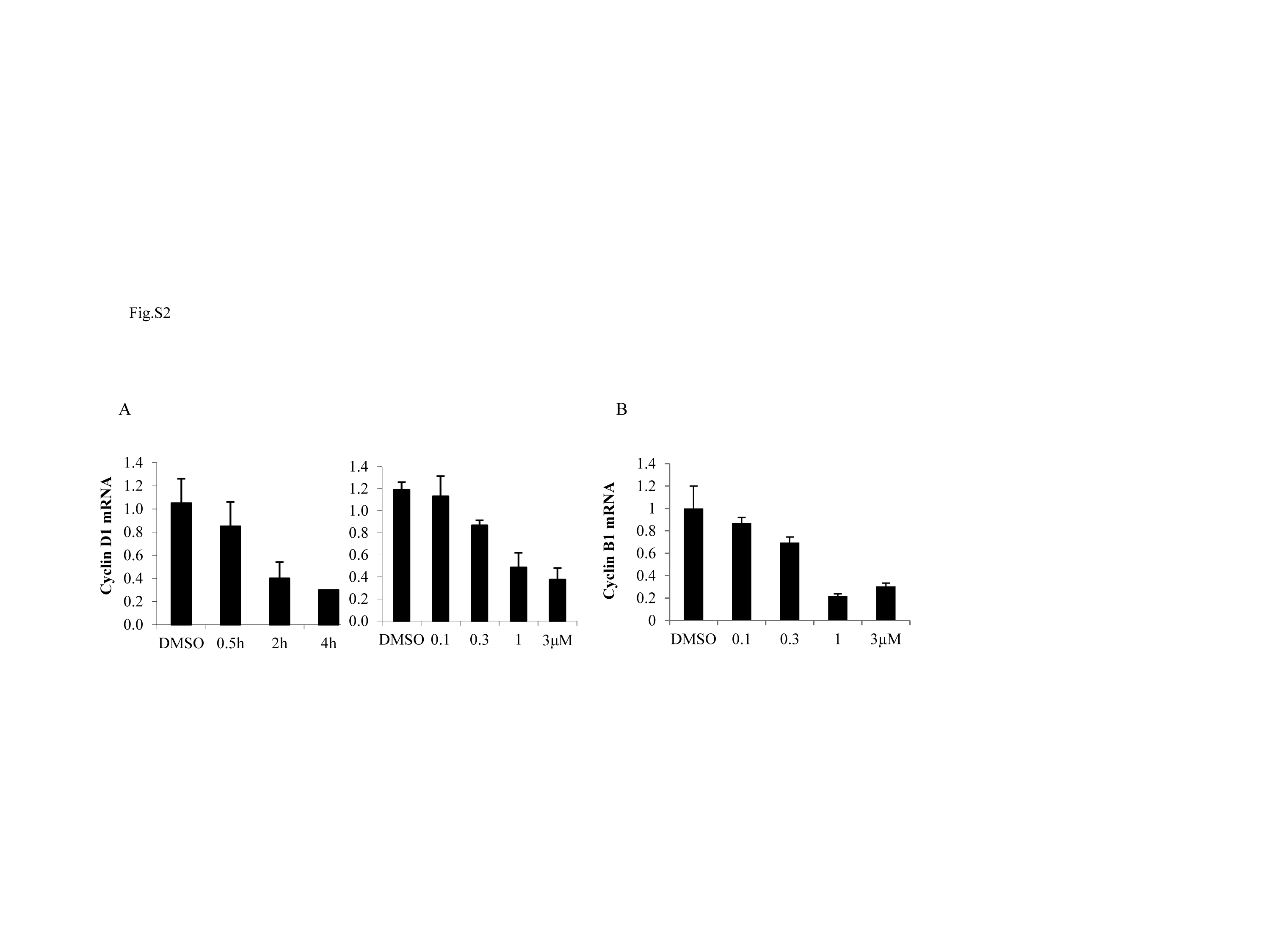

Supplement: Figure S2 — mRNA levels of Cyclin D1 and Cyclin B1 expression after treatment with NSC-743380. A) Time dependent and dose response changes of Cyclin D1 mRNA after treatment with NSC-743380 in A498 cells. B) Dose dependent changes of Cyclin B1 mRNA after treatment with NSC-743380 in A498 cells. The mRNA levels were normalized with that of GAPDH. (TIF) [file pone.0028487.s002.tif]

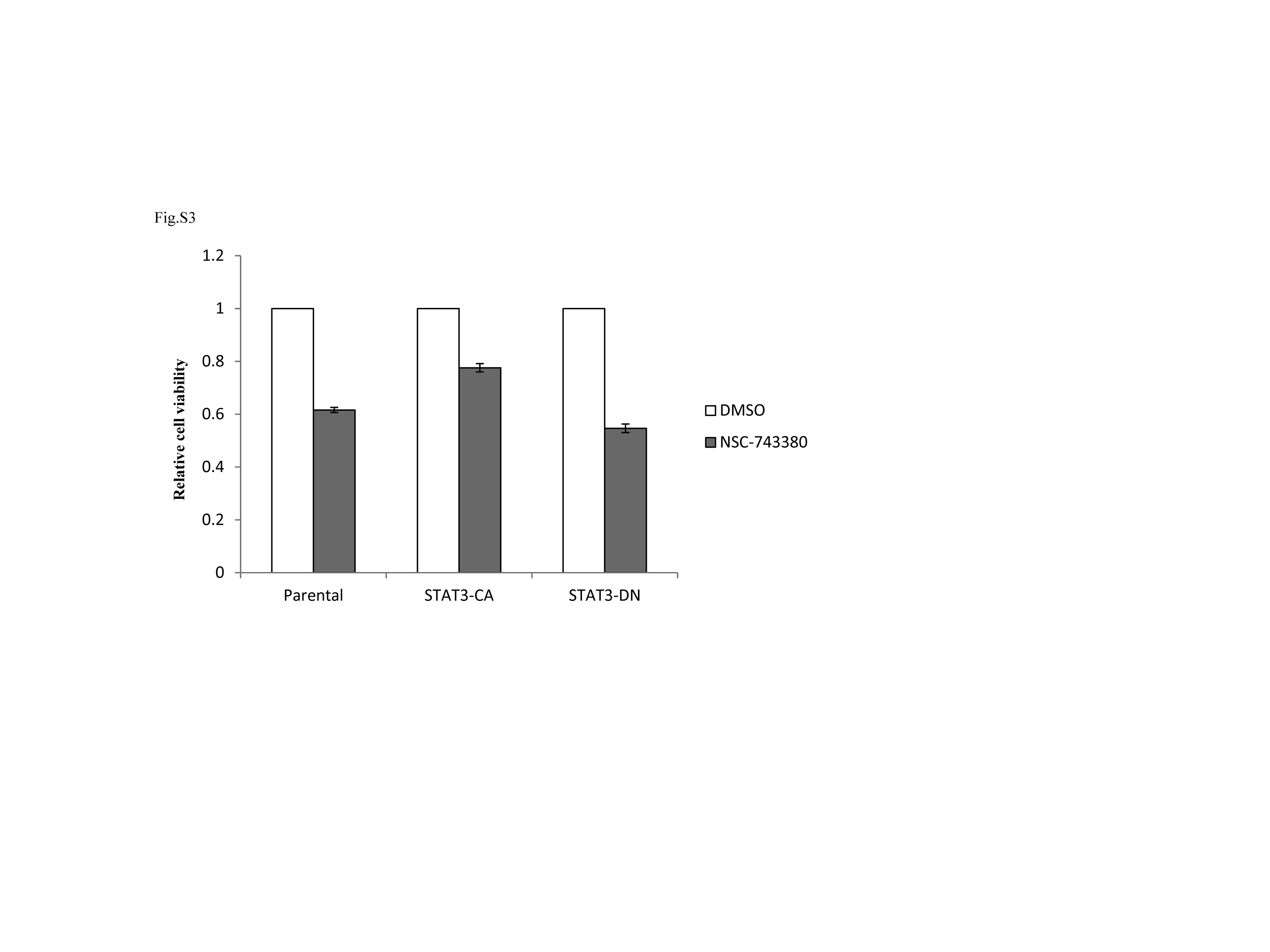

Supplement: Figure S3 — Cell viability of A498, A498 STAT3-CA and A498 STAT3-DN cells after treatment with 1 µM NSC-743380 for 12 h. Cells treated with DMSO were used as controls and set as 1. Cell viability was assayed 12 h after treatment by the SRB method. Each data point represents the mean ± SD of three independent experiments. (TIF) [file pone.0028487.s003.tif]
