# Supplementary material for: Antitumor Activity of a Novel Oncrasin Analogue Is Mediated by JNK Activation and STAT3 Inhibition
Source: PLoS One. 2011 Dec 12;6(12):e28487. doi: 10.1371/journal.pone.0028487 (PMC3236185; doi:10.1371/journal.pone.0028487)
Supplement: Text S1 — Method for synthesis, purity determination and structure identification of NSC-743380. (DOCX) [file pone.0028487.s004.docx]

**Text S1. Method for synthesis, purity determination and structure identification of NSC-743380**

NSC-743380 was synthesized as the following: 1H-indole-3-carboxaldehyde was reacted with 3-chlorobenzyl bromide in anhydrous dimethyl sulfoxide (DMSO) under alkali catalysis at room temperature to obtain intermediate product 1-[(3-chlorophenyl) methyl]-1H-indole-3-carboxaldehyde. After purification with silica gel chromatography, the intermediate product was reduced by NaBH_4_. The final product, 1-[(3-chlorophenyl) methyl]-1H-indole-3-methanol (NSC-743380), was purified by recrystallization with organic solvent ethyl ether/hexane. The purity and molecular weight of the compound were determined by high-performance liquid chromatography-mass spectrometry analysis. The purity was determined by HPLC (Agilent Technologies 1200 Series) equipped with a C-18 bounded-phase column (Waters, XTerra C18 MS, 3.5µm, 4.5 x 50mm). A gradient elution was performed with acetonitrile and water as a mobile phase and was monitored at 230nm. The compound used for activity study have purity of ≥ 98%. The chemical entities of compounds were determined by LC/MS/UV_230_ and ^1^H and ^13^C NMR analyses. Electrospray ionization (ESI) MS spectra were recorded with an Agilent LC/MSD Trap XCT Ultra spectrometer. NMR spectrum was recorded with Bruker Avance DRX 300 spectrometer as the following:

**NSC-743380.** ^1^H NMR (300 MHz, CDCl3): δ 7.78 (m, 1H, C4-indole), δ 7.23-7.15 (s,3H, indole and 4H, phenyl), δ 7.00 (m, 1H, C-2 indole), δ 5.28 (s, 2H, N-CH2), δ 4.91 (s, 2H, CH2-OH). ^13^C NMR (CDCl_3_, 75HMz): 141.2 (phenyl C-1), 136.7 (indole C-9), 133.6 (phenyl C-3), 130.8 (phenyl C-5), 127.8 (phenyl C-2, indole C-8), 127.3 (phenyl C-6, indole C-2), 126.3 (phenyl C-4), 121.8 (indole C-5), 119.8 (indole C-6), 119.2 (indole C-4), 116.5 (indole C-3), 110.2 (indole C-7), 55.7 (-CH2OH), 48.8 (N-CH2). ESI-MS m/z 254.1 [M+H-H2O].
