# Supplementary material for: Antitumor Activity of a Novel Oncrasin Analogue Is Mediated by JNK Activation and STAT3 Inhibition
Source: PLoS One. 2011 Dec 12;6(12):e28487. doi: 10.1371/journal.pone.0028487 (PMC3236185; doi:10.1371/journal.pone.0028487)
Supplement: Text S2 — Quantitative PCR for mRNA analysis. (DOCX) [file pone.0028487.s005.docx]

**Text S2. Quantitative PCR for mRNA analysis**

Total RNA was extracted from cells using TRIzol reagent ([Invitrogen](http://www.jbc.org/cgi/redirect-inline?ad=Invitrogen)). A 500-ng aliquot of each RNA sample was reverse-transcribed in a 20-μl reaction volume using the Taqman reverse transcription reagents (Applied Biosystems). The 10-fold dilutions of the cDNA product were used in real-time PCR analyses. Real-time PCR was carried out in 20 μl of a reaction mixture containing 2 μl of diluted cDNA, 10 μl of 2× Absolute Blue QPCR SYBR Green mix buffer (Thermo Fisher Scientific), 5.2 μl of double-distilled water, and 1.4 μl each of sense and antisense primers (70 nm final concentration). Real-time PCR assays were performed in triplicate using a CRF96^TM^ real-time system (Bio-Rad) with the following conditions: 95 °C for 15 min, 40 cycles at 95 °C for 10 s, 58 °C for 15 s, and 72 °C for 30 s. The following primer sequences for the genes were used: Cyclin D1, sense : 5’-TATTGCGCTGCTACCGTTGA-3’ antisense : 5’- CAATAGCAGCAAACAATGTGAAA-3’; Cyclin B1, sense :5’-CCATGGCGCTCCGAGTCACC-3’ antisense:5’-GGGCCGTAGGAACGCGTTT-3’ ;*GAPDH,* sense 5′- GGCTCTCCAGAACATCATCC-3′, and antisense 5′-TAGCCCAGGATGCCCTT-3′. The sets of gene primer for the target genes were confirmed to have amplification efficiency equal to that of the reference gene *GAPDH*. The relative RNA expression was calculated automatically by the installed software of the instrument with the ΔΔ*C_t_* method, using *GAPDH* as a reference gene.
